# Altered Circadian Timing System-Mediated Non-Dipping Pattern of Blood Pressure and Associated Cardiovascular Disorders in Metabolic and Kidney Diseases

**DOI:** 10.3390/ijms19020400

**Published:** 2018-01-30

**Authors:** Asadur Rahman, Arif Ul Hasan, Akira Nishiyama, Hiroyuki Kobori

**Affiliations:** 1 Department of Pharmacology, School of Medicine, International University of Health and Welfare, 4-2 Kozunomori, Narita, Chiba 286-8686, Japan; rahmanma@iuhw.ac.jp (A.R.); hasan@iuhw.ac.jp (A.U.H.); 2 Department of Pharmacology, Faculty of Medicine, Kagawa University, Kagawa 761-0793, Japan; akira@med.kagawa-u.ac.jp

**Keywords:** blood pressure circadian rhythm, circadian timing system, metabolic disorders, chronic kidney disease, cardiovascular disease

## Abstract

The morning surge in blood pressure (BP) coincides with increased cardiovascular (CV) events. This strongly suggests that an altered circadian rhythm of BP plays a crucial role in the development of CV disease (CVD). A disrupted circadian rhythm of BP, such as the non-dipping type of hypertension (i.e., absence of nocturnal BP decline), is frequently observed in metabolic disorders and chronic kidney disease (CKD). The circadian timing system, controlled by the central clock in the suprachiasmatic nucleus of the hypothalamus and/or by peripheral clocks in the heart, vasculature, and kidneys, modulates the 24 h oscillation of BP. However, little information is available regarding the molecular and cellular mechanisms of an altered circadian timing system-mediated disrupted dipping pattern of BP in metabolic disorders and CKD that can lead to the development of CV events. A more thorough understanding of this pathogenesis could provide novel therapeutic strategies for the management of CVD. This short review will address our and others’ recent findings on the molecular mechanisms that may affect the dipping pattern of BP in metabolic dysfunction and kidney disease and its association with CV disorders.

## 1. Introduction

Blood pressure (BP) usually follows a circadian pattern of oscillation over a 24 h period with a decline of 10–20% at nighttime, and this normal pattern is referred to as the dipping pattern of BP [1]. The loss of this nocturnal decline, i.e., the development of a non-dipping type of BP, is frequently observed in metabolic disorders and chronic kidney disease (CKD) and contributes to the development of cardiovascular (CV) disease (CVD) [2]. A number of intrinsic and extrinsic factors influence the dipping pattern of BP. Several recent studies focusing on chronobiology have revealed the critical role of the circadian timing system in the regulation of BP in pathophysiological conditions (reviewed in [3,4,5,6]). A more thorough understanding of how the circadian timing system is related to the non-dipping pattern of BP in metabolic dysfunction and CKD would be helpful in developing novel therapeutic strategies for preventing the onset and development of CVD. Therefore, in this review, we summarize the recent findings on the molecular mechanisms related to the involvement of an altered circadian timing system in the development of the non-dipping pattern of BP and subsequent CVD in subjects with metabolic dysfunction and CKD. In addition, we discuss currently available and possible future chronotherapeutics that may be useful for patients with metabolic disorders and kidney disease.

## 2. Circadian Timing System and BP Regulation

**Figure 1:**
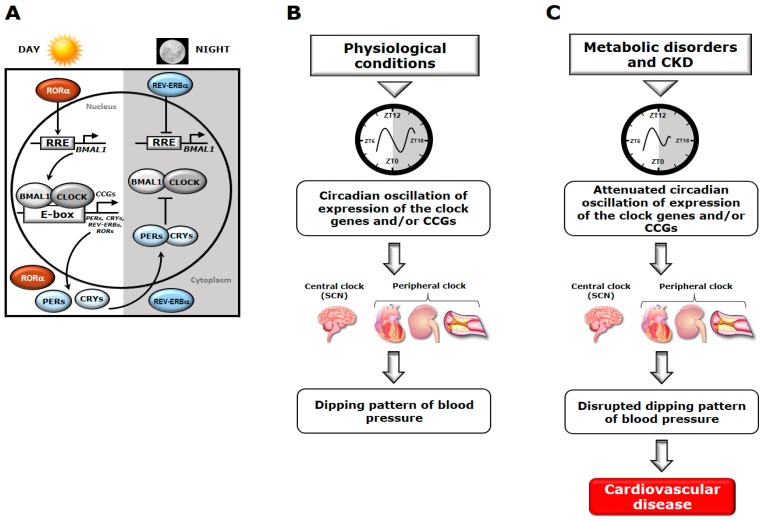
Schematic representation of the molecular components of the circadian timing system (**A**), and the status of circadian oscillation in the expression of clock genes and/or clock-controlled genes (CCGs) under physiological conditions (**B**) and in metabolic disorders and chronic kidney disease (CKD) (**C**). Under physiological conditions, the expression levels of the clock genes and/or CCGs undergo circadian oscillation in the central (suprachiasmatic nucleus [SCN] of the brain) and peripheral (e.g., heart, kidney, vasculature) clocks, mediated by transcriptional and translational feedback loops. The integral components of the core loop, CLOCK and BMAL1, form a heterodimer that induces E-box–mediated transcription of the negative regulators known as *PERs* and *CRYs*. Following accumulation of PER and CRY proteins, E-box–mediated transcription is repressed until cleared by proteasomal degradation. By contrast, in the regulatory or stabilizing loop, RORα and REV-ERBα modulate the *BMAL*1 mRNA levels by competitive actions on the REV-ERB/ROR–responsive element (RRE) residing in the *BMAL*1 promoter. All these components comprising the core and regulatory clocks determine the expression levels of core clock genes and/or CCGs via the E-box and/or RRE. Therefore, a rhythmic expression pattern is generated in physiological processes; regarding blood pressure (BP), for instance, this pattern is referred to as a dipping pattern. In subjects with metabolic dysfunction and CKD, however, loss of the circadian oscillation in the expression of core clock genes and/or CCGs leads to a disrupted dipping pattern of BP, which inevitably plays a critical role in the development of cardiovascular disorders. BMAL1: Brain and muscle arnt-like 1; CLOCK: Circadian locomotor output cycles kaput; CRYs: Cryptochromes; PERs: Periods; REV-ERVs: Nuclear receptors encoded by nuclear receptor subfamily 1, group D (NR1D); RORs: Retinoic acid receptor-related orphan receptors; RRE: REV-ERB/ROR responsive element; ZT: Zeitgeber time.

The circadian regulation of BP is influenced by a number of extrinsic (light and food) and intrinsic (physiological and biological) factors [7]. One intrinsic factor is the circadian timing system, which is known to govern the circadian oscillation of BP [8] by its central and/or peripheral clocks. The circadian rhythm of BP was previously thought to be regulated primarily by the central clock, which is a unique circadian pacemaker residing in the suprachiasmatic nucleus (SCN) of the hypothalamus of the brain [7]. However, recent advanced studies have demonstrated the presence of circadian clocks in peripheral tissues, especially in the heart, vasculature, kidneys, and neuroendocrine system, which also contribute considerably to the circadian regulation of BP [3]. At the molecular level, both the central and peripheral clocks are composed of a core of interconnected transcriptional and translational oscillating regulatory feedback loops (reviewed in [9], Figure 1A). During the daytime, the transcription factor brain and muscle Arnt-like protein 1 (BMAL1), a core component of the circadian timing system, forms a heterodimer with circadian locomotor output cycles kaput (CLOCK), which induces E-box (CACGTG)–mediated transcription of repressor clock genes, namely *PERIODS* (*PER*1, 2, and 3) and *CRYPTOCHROME* (*CRY*1 and 2). Following translation, PERs and CRYs interact with each other, translocate into the nucleus, and repress E-box–mediated transcription by inhibiting the activity of the CLOCK-BMAL1 heterodimer. In the absence of de novo synthesis, PERs and CRYs are progressively depleted during the nighttime by the ubiquitination or proteasomal degradation pathway, thus resulting in the termination of the transcriptional repression and reinitiation of a new cycle of the transcription–translation feedback loop [9,10]. In addition to this core loop, an auxiliary or stabilizing feedback loop forms a reinforcing mechanism comprised of clusters of circadian nuclear receptors, namely retinoic-acid-receptor–related orphan nuclear receptors (RORs; RORα, β, and γ) and REV-ERBs (REV-ERBα and β, encoded by *NR*1*D*1 and *NR*1*D*2 genes, respectively), which are also under the transcriptional control of the CLOCK-BMAL1 complex. REV-ERBs and RORs compete with each other to bind with REV-ERB/ROR–responsive elements (RREs) residing in the *BMAL*1 promoter. In this regulatory loop, RORα transcriptionally activates *BMAL*1 mRNA expression, whereas REV-ERBα strongly suppresses it [11,12]. Collectively, cycling of these molecular components of the circadian timing system determines the levels of various clock-controlled genes (CCGs) in the SCN and/or peripheral tissues (kidneys, vasculature, and heart) by transcription via E-box and/or RREs, thus generating rhythmic physiological outputs. Witte et al. (1998) observed that destruction of the SCN in both normotensive and hypertensive rats abolished the dipping pattern of BP, suggesting a critical role of the SCN in BP regulation [13]. Moreover, the expression levels of the core clock genes (*CLOCK*, *BMAL*1, *CRYs*, and *PERs*) as well as their target genes (about 8% to 10% of the total genes) in the peripheral organs including the kidneys, heart, and vasculature undergo dramatic circadian oscillation [14], which is essential for maintaining the diurnal variation of BP under physiological conditions (Figure 1B).

## 3. The Circadian Timing System and Diurnal Variation of BP in Metabolic Dysfunction

Disruption of the circadian rhythm of BP is associated with a significantly worse prognosis in subjects with metabolic abnormalities [15], even in the absence of hypertension [16]. Moreover, when hypertension is superimposed on a metabolic disorder, the progression of pathological complications becomes significantly more severe. Substantial advances have been made over the past decade in our understanding regarding the powerful effect of an altered circadian timing system in the development of the disrupted dipping pattern of BP in subjects with metabolic dysfunction (Table 1). Evidence from an experimental animal model of type 2 diabetes (T2D, *db*/*db* mice) indicated a severely disrupted dipping profile of BP [17]. Furthermore, the pathological manifestations associated with metabolic abnormalities are accompanied by altered diurnal variations in the expression of clock genes in both the central and peripheral clocks, as well as their target genes [18,19,20].

### 3.1. Glucose Homeostasis and Insulin Function

Clinically, postprandial hyperglycemia is associated with a disrupted circadian rhythm of BP in subjects with T2D [27]. Interestingly, a study using knockout (KO) mice revealed that inactivation of *Bmal*1 and *Clock* suppresses the diurnal variation in glucose and triglyceride levels and eliminates gluconeogenesis, signifying the critical roles of core clock genes in glucose homeostasis. Of note, Hsieh et al. (2010) demonstrated that hyperglycemia in mice with high-fat-diet–induced obesity was associated with alteration of almost all circadian core clock genes, as well as their target genes, namely albumin-D-site-binding protein (*Dbp*), phosphoenolpyruvate carboxykinase (*Pepck*), and pyruvate dehydrogenase kinase-4 (*Pdk*4), which are involved in the circadian transcriptional regulation of several metabolic enzymes, gluconeogenesis, and lipolysis, respectively [19].

Disruption of the circadian timing system resulted in a significant decrease in glucose-stimulated insulin secretion [28], while disruption of the core clock components *Clock* and *Bmal*1 led to hypoinsulinemia and diabetes [29]. Clinically, insulin resistance also plays a pivotal role in the pathogenesis of T2D with a non-dipping pattern of BP [30] and subsequent CV complications [31]. By contrast, circadian-clock-disrupted *Bmal*1-KO mice [32] and homozygous *Clock*-mutant mice [33] exhibit hyperglycemia and insulin resistance, as well as obesity. When rhythmicity is rescued in *Bmal*1-KO mice by expression of the paralogous gene *Bmal*2, insulin sensitivity is restored. Furthermore, the increased expression of *Pepck* and *Pdk*4 genes (CCGs in the kidneys and liver) with higher levels of blood glucose and serum insulin exacerbated the processes of insulin resistance and diabetes in high-fat-diet-induced obesity [19]. All of these data indicate the critical roles of altered expression patterns of core clock genes and their target genes in the pathogenesis of metabolic abnormalities and obesity, which are closely associated with the development of a non-dipping pattern of BP.

### 3.2. Cholesterol and Triglycerides

A clinical study by Mergeani et al. (2015) demonstrated that increased total and low density lipoprotein (LDL) cholesterol are significantly associated with a disrupted circadian rhythm of BP [34]. Interestingly, mice lacking functional LDL-receptor, an animal model of human familial hypercholesterolemia, exhibit abnormal circadian behavior [35]. Additionally, the introduction of a genetic mutation in the *Per*2 gene resulted in mice with a significant enlargement of artery plaque area and increased levels of the inflammatory cytokine interleukin 6 (IL-6). Another clinical study has likewise demonstrated that early atherosclerosis is associated with a non-dipping pattern of BP [36]. Moreover, nuclear receptor *Rev-erb*α deficiency in mice leads to hypercholesterolemia and enhances the development of atherosclerosis [37]. In addition, abnormally high levels of serum cholesterol, altered diurnal rhythm in serum triglycerides and free fatty acids, and altered regulation of adipocyte differentiation have been demonstrated in *Clock*-mutant mice [38,39].

Plasminogen activator inhibitor (PAI)-1 is positively correlated with triglyceride levels in subjects with diabetes [40] and is significantly higher in subjects with a non-dipping pattern of BP [41]. In this regard, Kudo et al. (2004) demonstrated that significantly increased *Pai-*1 mRNA expression in the liver was associated with altered rhythmic expression of the core clock genes *Per*2 and *Bmal*1 in *db*/*db* mice [20]. Furthermore, levels of plasma PAI-1 were significantly increased in streptozotocin (STZ)-induced diabetes in *Clock*-mutant mice, leading to diminished circadian oscillation of *Pai*-1 [21]. This suggests that the molecular components of the circadian timing system play a critical role in the development of metabolic abnormalities and the consequent further attenuation of the dipping pattern of BP.

### 3.3. Endothelial Function and Vascular Tone

The circadian oscillation of clock genes and their target genes is markedly disrupted not only in metabolic organs but also in the vasculature of subjects with diabetes. Su et al. (2008 and 2012) demonstrated that *db*/*db* mice exhibited a disrupted circadian rhythm of BP [17] and altered diurnal contractile variations of vascular smooth muscle cells (VSMCs) in the aorta and mesenteric arteries [18] due to the disrupted circadian oscillation of contraction regulatory proteins, namely Rho-associated protein kinase 1 (ROCK1) and protein kinase C-potentiated phosphatase inhibitory protein (CPI-17) [18]. It has also been demonstrated that, following binding with a promoter, *Bmal*1 activated the transcription of *Rock*2 in mesenteric arteries, and *Bmal*1 deletion abolished diurnal variations in response to agonist-induced vasoconstriction, myosin phosphorylation, and ROCK2 activation [42]. Moreover, systemic deletion of *Bmal*1 also caused endothelial dysfunction and vascular stiffness due to attenuated Akt and nitric oxide signaling [43], increased expression of the homeostasis-related glycoprotein Von Willebrand factor (vWF) [44], increased production of reactive oxygen species (ROS) by superoxides and uncoupling of nitric oxide synthase [45,46], and dysfunction of matrix metalloproteinase (MMP) 2 and 9 [47].

Peroxisome proliferator-activated receptor (PPAR)-γ is a nuclear receptor and transcription factor that plays critical roles in the vasculature by suppressing inflammatory gene expression and therefore improves endothelial dysfunction in subjects with metabolic abnormalities [48]. By contrast, *Ppar*-γ deletion in the aorta resulted in significant alteration in the diurnal rhythm of BP along with disrupted circadian oscillation of *Bmal*1 [49], suggesting a critical role of the *Bmal*1 gene in the maintenance of vascular function. Anea et al. (2009) likewise demonstrated that mutation in the *Clock* gene induced endothelial dysfunction that was associated with the attenuation of Akt signaling and a subsequent decrease in nitric oxide production [38]. Moreover, mutation in the *Per*2 gene is associated with impaired endothelial dysfunction due to increased expression of cyclooxygenase [50] and increased Akt signaling [51]. A phenotype similar to that seen in this form of endothelial dysfunction is observed in mice with deletion of all the Period gene isoforms (*Per*-triple KO mice) [47]. Lin et al. (2014) demonstrated that plaque-derived human VSMCs exhibit altered rhythmic expression of the core clock genes *BMAL*1, *CLOCK*, *PER*2, and *REV-ERB*α [52]. Moreover, enhanced lipid accumulation in the aorta is also observed in mice deficient in the nuclear transcription factor *Rev-erb*α, which triggers atherosclerosis through the increased expression and secretion of the pro-atherogenic cytokine IL-18 in primary macrophages and the increased expression of *Nlrp*3, an inflammasome component involved in IL-18 maturation [37]. Together, these data suggest that altered expression of the molecular components of the circadian timing system is associated with the development of endothelial dysfunction and vascular stiffness in subjects with metabolic diseases, and that this process may play a vital role in developing a disrupted circadian rhythm of BP.

### 3.4. Autonomic Nervous Function

Among the several signaling pathways involved in the regulation of the circadian rhythm of BP, the central clock in the SCN plays a critical role by transmitting sympathetic and parasympathetic signals to the heart, kidneys, and vasculature [53]. More specifically, oscillation of sympathetic activity evidently regulates the circadian oscillation of BP. Mounting evidence indicates that the circadian rhythm of sympathetic nervous function is disrupted in patients with metabolic abnormalities [54] and associated CVD [55]. We have also demonstrated that a non-dipping pattern of BP is closely associated with altered diurnal variation of sympathetic nervous function in animal models of metabolic abnormalities [56]. Recent studies have shown that a disrupted circadian rhythm of BP is closely associated with altered catecholamine (dopamine, norepinephrine, and epinephrine) levels [49,57]. Systemic deletion of *Bmal*1 alters the diurnal variation of catecholamines by altering the enzymes associated with synthesis and disposition, leading to altered diurnal variation of BP [58]. Moreover, under a light-dark cycle, destruction of the SCN attenuated the daily rhythms of not only *Per*1, *Per*2, and *Bmal*1 gene expression, but also nor-adrenergic activity, whereas the administration of catecholamines restored normal oscillations of *Per*2 and *Bmal*1 gene expression in the liver of mice with SCN lesions [59]. These data are consistent with the hypothesis that alteration of central clock activity is associated with impaired autonomic nervous function, which in turn permits the development of a non-dipping BP pattern in subjects with metabolic abnormalities.

### 3.5. Sleep Disturbances

It is well accepted that the diurnal variation of physiological processes is important for health, and that there is a strong link between sleep disturbance, impaired circadian rhythm, metabolic disorders, and related CV disorders. Recent clinical studies have demonstrated that nightshift work is associated with the development of metabolic syndrome [60] accompanied by the development of a non-dipping type of hypertension [61]. A study involving experimental disruption of the light-dark cycle in the form of either an advancing light period or a constant light period in diabetes-prone human islet amyloid polypeptide transgenic (HIP) rats accelerated the development of diabetes by increasing the loss of beta-cell function [62]. Indeed, mice exposed to dim light at night exhibit attenuated amplitudes of *Per*1 and *Per*2 rhythms in the hypothalamus and *Rev-erb* expression in the liver and adipose tissues, which promotes metabolic abnormalities and obesity [22] and a consequent development of a non-dipping pattern of BP.

### 3.6. Genetic Involvement

Genome-wide association studies (GWASs) have indicated a genetic link between single nucleotide polymorphisms (SNPs) in circadian core clock genes and metabolic abnormalities [63]. Genetic variants in circadian genes are also associated with the non-dipping pattern of hypertension, suggesting a genetic association with diurnal variation of BP [64]. Woon et al. (2007) performed a landmark study on the genetic variations of *BMAL*1 and their association with hypertension and diabetes [23]. Following a functional analysis of rat *Bmal*1 promoter polymorphisms and based on the candidate SNPs, the authors performed translational research in humans, which revealed a strong association of *BMAL*1 SNPs with hypertension (three-marker haplotypes; rs6486121, rs3789327, rs969485) and diabetes (two-marker haplotypes; rs7950226, rs11022775) in humans, although the associated SNPs were not from the same haplotypes. Genetic variation in the *CLOCK* gene was associated with the development of T2D [65]. Furthermore, Corella et al. (2016) detected a statistically significant interaction between *CLOCK* gene variation (*CLOCK*-rs4580704 SNP, C > G) and stroke in subjects with T2D [24]. Variation in *CRY* (*CRY*1 and 2) genes was also associated with susceptibility to T2D [66,67], while *CRY*1 polymorphism increased the risk of systemic insulin resistance in the presence of high carbohydrate intake [68]. Furthermore, Sookoian et al. (2010) demonstrated a significant interaction between polymorphisms in the promoter region of the serotonin transporter gene and common variants of the *CLOCK* gene affecting diastolic, systolic, and arterial hypertension, as well as plasma triglyceride levels in rotating shift workers, who are particularly susceptible to developing metabolic abnormalities and a non-dipping pattern of BP [25]. Although these results indicate that genetic variations in circadian core clock genes are closely associated with the development of hypertension and metabolic abnormalities, more extensive studies will be required to improve our understanding of the relationship between genetic variations in core clock genes and the development of a non-dipping pattern of BP and associated CV events.

## 4. The Circadian Timing System and Diurnal Variation of BP in CKD

Hypertension is often accompanied by CKD, and its incidence and prevalence increase over the course of CKD progression to end-stage renal disease (ESRD) [69]. Moreover, a disrupted dipping pattern of BP, especially a non-dipping profile, is a common finding among patients in the early stages of CKD and is predictive of CVD [70,71]. Accumulating evidence has pointed out the critical role of the molecular components of the circadian timing system in the development of a non-dipping pattern of BP in subjects with CKD (Table 2). Recent molecular dissection of the circadian timing system has revealed that the peripheral clocks, especially those in the vasculature and kidneys, are severely disrupted in CKD; this condition may be involved in the pathogenesis of the non-dipping pattern of BP in subjects with CKD.

### 4.1. Renal Function

Goto et al. (2005) [78] demonstrated that a decrease in creatinine clearance induced by unilateral nephrectomy (kidney donation) was positively correlated with an increase in the night/day (N/D) ratio of BP, although it was not correlated with changes in mean 24-h BP. These findings suggest that unilateral nephrectomy itself can disturb the dipping pattern of BP by interfering with renal function without affecting absolute levels of BP. In keeping with this, circadian BP variation was markedly impaired in patients in the earliest stages of CKD, whose kidney function was assessed by proteinuria or estimated glomerular filtration rate. Huang et al. (2013) demonstrated that the localization and circadian variation of certain clock proteins, namely BMAL1, DBP, and PER2, which are involved in the diurnal rhythm of renal function, were altered in rats with subtotal nephrectomy [72]. Therefore, it can be postulated that impairment of the circadian timing system may be associated with the development of a non-dipping pattern of BP in subjects with CKD.

### 4.2. Endothelin and Renin Angiotensin System

In a study by Dhaun et al. (2014), nighttime decreases in BP were abolished and the endothelin system was significantly upregulated in subjects with CKD compared to healthy volunteers, suggesting a strong association between endothelin expression and a non-dipping profile of BP [79]. Importantly, endothelin expression is regulated by the circadian clock protein PER1 in the kidneys in a time-dependent manner, and it has been shown that altered *Per*1 expression may affect the endothelin system as well [80]. By contrast, the endothelin system is closely involved with the renin angiotensin system (RAS) [81], and RAS hyperactivity is associated with the progression of CKD to ESRD [82,83]. By measuring the N/D ratio of urinary angiotensinogen, we and others have shown that the circadian rhythm of intrarenal RAS activation in subjects with CKD may lead to renal damage and hypertension [84,85], while Isobe et al. (2015) have demonstrated that the N/D ratio of urinary angiotensinogen is positively correlated with the N/D ratios of urinary protein, albumin excretion, and BP [84], suggesting that activation of the intrarenal RAS plays a critical role in the pathophysiology of the dipping pattern of BP. Tissue-specific regulation of the expression of the angiotensin II type 1 receptor (AT1R)-associated protein (ATRAP/Agtrap) is relevant to the pathophysiology of renal and CV diseases, because it promotes internalization of AT1R. Matsuda et al. (2013) postulated that unilateral ureteral obstruction (UUO) leads to a decrease in *Agtrap* mRNA expression, which negatively regulates angiotensin II signaling in the afflicted kidney [73]. Moreover, they found that *Agtrap* is decreased by upstream stimulatory factor (Usf) 1, a known E-box–binding transcription factor, which is an important modulator of molecular and behavioral circadian rhythms in mammals [86]. Therefore, these data strongly suggest an involvement of the molecular components of the circadian timing system in the impairment of endothelin and the RAS system, with subsequent development of a non-dipping pattern of BP in subjects with CKD.

### 4.3. Water and Electrolyte Homeostasis

Appropriate water and electrolyte balance is a key determinant of BP [87], and impaired homeostasis is linked to the non-dipping pattern of hypertension and accelerated progression of CKD [88]. Huang et al. (2013) postulated that the diurnal expression of circadian clock genes (*Bmal*1, *Per*2, and *Dbp*) in the kidney was significantly altered in association with the diurnal rhythm of BP in rats with subtotal (5/6) nephrectomy [74]. Importantly, Tokonami et al. (2014) demonstrated that kidney-specific deletion of *Bmal*1 led to an imbalance in water and electrolyte homeostasis and a marked reduction in plasma aldosterone levels accompanied by a decrease in BP [89] due to alteration of the circadian oscillation of *Ren*1, *Cyclooxygenase*-2, and *Ppar*-γ. Since these genes are involved in water and electrolyte homeostasis, the study results support a powerful role of BMAL1 in the dipping pattern of BP. Similarly, systemic deletion of *Clock* led to a decrease in BP through alteration of the renal expression of key regulators of water and/or sodium balance [90,91]. Furthermore, *Clock* deficiency causes the loss of the circadian expression of estrogen-related receptor β, which is closely associated with the expression of Na-K-Cl cotransporter type 2 (NKCC2) and the regulation of potassium and sodium homeostasis [92]. Moreover, Saifur Rohman et al. (2005) demonstrated that BMAL1 and CLOCK together regulate the circadian oscillation of Na^+^/H^+^ exchanger 3 (NHE3) [93], which regulates sodium homeostasis in the kidney and contributes to the regulation of BP [94,95]. Silencing of *Per*1 in proximal tubular cells reduced the expression of SGLT1 and NHE3 [96], while silencing of *Per*1 in collecting duct cells reduced the expression of the alpha subunit of the epithelial Na^+^ channel (ENaCα) [97], suggesting an important role of *Per*1 in the regulation of sodium transport. Consistently, mice lacking functional *Per*1 exhibit a dramatic reduction in BP which is associated with decreased expression of ENaCα [98] and 3-(β)-hydroxysteroid dehydrogenase (3β-HSD, an enzyme involved in aldosterone synthesis) [99], as well as increased endothelin-1 expression in the kidney [100]. Moreover, the absence of a nocturnal fall in BP is commonly seen in CKD patients with salt-sensitive hypertension [101]. In an experimental rat model, animals with adenine-induced chronic renal failure also exhibited salt-sensitive hypertension due to sodium retention as well as elevated aortic stiffness following renal insufficiency [102]. An outstanding study by Doi et al. (2010) postulated that mice deficient in the core clock components *Cry*1 and *Cry*2 showed salt-sensitive hypertension due to the increased production of aldosterone by the adrenal gland [103]. They also found that the expression of β-hydroxyl-steroid dehydrogenase (Hsd3b6) exclusively in aldosterone-producing cells led to a marked increase in 3β-HSD enzymatic activity and, as a consequence, enhanced aldosterone production in *Cry*-deficient mice. Interestingly, Hsd3b6 is also under transcriptional control of the circadian clock. Gumz et al. (2010 and 2009) demonstrated that aldosterone upregulates the expression level of ENaCα [97,98]. Casein kinases 1δ and 1ε (CK1δ/ε) are required for the regulation of PER/CRY heterodimers, and it has been postulated that inhibition of these casein kinases reduces the expression and activity of ENaCα [104]. Together, all of these data clearly indicate a strong association between the molecular components of the circadian timing system and the pathogenesis of water and electrolyte imbalances. These data also suggest that the alteration of these components may lead to a disrupted dipping pattern of BP in subjects with CKD.

### 4.4. Renal Fibrosis

A disrupted circadian rhythm of BP is significantly associated with renal tissue injury and renal tubulointerstitial fibrosis [105]. It has been reported that 5/6 nephrectomy in wild-type mice caused aggravated renal fibrosis and severely disrupted the expression profile of circadian core clock genes [75]. Plasma transforming growth factor-β1 (TGF-β1) is also significantly increased in mice with 5/6 nephrectomy, leading to decreased expression of *Dbp*, which controls the expression of *Cyp*3*a*11 and *Cyp*26*a*1 in the liver and is involved in retinol metabolism. These findings suggest that the alteration of the circadian clock in the liver–kidney axis aggravates renal dysfunction. In keeping with these findings, Chen et al. (2015) have demonstrated that UUO in *Clock*-deficient mice led to increased renal fibrosis and injury due to increased mRNA expression of *Tgf*-β and *Cyclooxygenase* 2 [76]. It has also been shown that *Tgf*-β mRNA diurnal oscillation is controlled by CLOCK-BMAL1 heterodimers [106]. All of these findings suggest that the molecular components of the circadian timing system are involved in the development of tubulointerstitial fibrosis, which in turn is linked to the development of an abnormal circadian rhythm of BP in CKD.

### 4.5. Melatonin

The nocturnal increase in endogenous melatonin, which is associated with the onset of sleep propensity, is absent in hemodialysis patients [107]. As melatonin amplitude and melatonin rhythm decrease with advancing renal dysfunction, follow-up research on the circadian timing system in patients with CKD is warranted. Hsu et al. (2012) have shown that both slow wave sleep and rapid eye movement sleep were significantly increased during dark periods in rats with subtotal nephrectomy, compared with the sleep patterns of control rats [77]. These CKD-induced sleep disruptions are associated with significant upregulations of *Per*1 and *Per*2 mRNA expression in the hypothalamus, suggesting that a disrupted circadian timing system plays a role in the pathogenesis of sleep disturbance in patients with CKD, which is closely associated with the development of an attenuated dipping pattern of BP.

## 5. Altered Circadian Timing System and Disrupted Dipping Pattern of BP in the Development of CV Disorders

A non-dipping pattern of BP is greatly associated with the onset and development of CVD, such as myocardial infarction, stroke, angina, and heart failure. These clinical events have been attributed primarily due to alteration of environmental cues (light or food) and/or neurohormonal changes (increased RAS or sympathetic-nerve activity) in metabolic disorders and CKD [6,8]. Melatonin is an endogenous product of the pineal gland that acts as a messenger of the SCN and thereby plays an important role in synchronizing CV functions [108,109]. Interestingly, subjects with the non-dipper type of hypertension express a lower nocturnal surge of melatonin release, as revealed by reduced 6-sulfametoxymelatonin in the urine [110] and a lower N/D ratio of melatonin concentration [111]. Moreover, a strong correlation has been observed between plasma levels of melatonin and the severity of CVD; specifically, greater reductions in melatonin production are observed in patients at higher risk of myocardial infarction and/or sudden death [108]. Through diverse forms of evidence from chronobiology studies, however, it is becoming increasingly clear that an impaired circadian timing system is likely associated with CVD (reviewed in [5,112,113]). It has been shown that exogenous melatonin administration changes the circadian oscillation of core clock genes [114] and modulates the effects of nuclear orphan receptor REV-ERBα [115] in the SCN, thereby modulating CV functions. 

More importantly, alteration of the molecular components of the circadian timing system is associated with disruption of the circadian rhythm of BP. Cardiomyocyte-specific *Bmal*1-KO mice exhibit dilated cardiomyopathy with prolonged and diminished circadian oscillation of CV function [116]. The precise molecular mechanism underlying this pathology has not been completely elucidated. However, recent studies have demonstrated that the deletion of *Bmal*1 from cardiomyocytes leads to the disruption of the circadian rhythms of *Scn*5*a* [117] and *Kcnh*2 [118], which are responsible for maintaining the sodium ion current and potassium channel, respectively, in ventricular myocytes. Several studies have postulated possible molecular mechanisms by which dilated cardiomyopathy results from the deletion of *Bmal*1, including increased expression of collagen (I and III) and MMP 9, 13, and 14 [116], increased expression of molecular marker for hypertrophy (*Mcip*1) [119], and altered circadian oscillation of a number of genes associated with myosin heavy chain [120], cardiac metabolism, and the insulin signaling cascade [120].

CLOCK comprises the other part of the activated heterodimer with BMAL1. Cumulative data from cardiomyocyte-specific *Clock*-mutant mice have revealed a phenotype (hypertrophic cardiomyopathy with prolonged heart rate) similar to that of *Bmal*1-KO mice. The pathology of *Clock-*mutant mice is also associated with increased expression of hypertrophy markers (*Anf* and *Mcip*1) [119] and altered diurnal variation of gene expression related to metabolism in the atria and ventricles [121]. Overexpression of *Clock-Bmal*1 in cardiomyocytes causes decreased expression of the L-type calcium channel and increased PI3-Akt signaling, suggesting a regulatory role of CLOCK-BMAL1 in cardiac electrogenesis and arrhythmogenesis [122].

By contrast, Wang et al. (2010) demonstrated that deletion of all three Par bZip transcription factors (*Dbp*/*Tef*/*Hlf*) also caused hypertrophic cardiomyopathy associated with increased sympathetic-nerve activity, decreased aldosterone production, and subsequent decrease in BP [123].

The alteration of the circadian expression of core clock genes and/or CCGs may perturb the adaptive response of cardiomyocytes to external stimuli and accelerate hypertrophic processes, leading to the development of CV disorders.

In rats with metabolic abnormalities [26] and CKD [72], the phases of circadian oscillation of the molecular components of the circadian timing system are altered. This phase alteration impairs synchronization with environmental cues, which may in turn cause loss of synchronization between the central and/or the peripheral clocks (clock and CCGs) by neurohormonal stimulation. In terms of clinical translation, the phase shift of the molecular components of the circadian timing system or the loss of synchronization may contribute to the development of a disrupted dipping pattern of BP in subjects with metabolic dysfunction and CKD, which subsequently exacerbates CV disorders (Figure 1C). 

## 6. Circadian Timing System-Based Therapies for the Non-Dipping Pattern of Hypertension in Metabolic and Kidney Diseases

Disruption of the circadian rhythm of BP, particularly disruption of the nighttime drop in BP, in metabolic dysfunction and/or CKD is an important predictor of further development of CVD. Recent data from animal and clinical studies have revealed that treatment with a sodium-dependent glucose cotransporter (SGLT) 2 inhibitor improves CV outcomes and mortality, and that the cardioprotective effects of SGLT2 inhibitors are associated with a reduction in BP, as well as normalization of the dipping pattern of BP from the non-dipper to the dipper type in subjects with metabolic abnormalities [56,124,125,126]. We also demonstrated that intervention with an angiotensin II type 1 receptor blocker with or without hydrochlorothiazide improves nocturnal hypertension and slows the progression of renal injury in patients with CKD to ESRD with CV complications [127,128]. Taken together, the findings of these pharmacological investigations suggest that normalization of the circadian rhythm of BP is crucial to prevent the development of CVD in subjects with metabolic dysfunction and CKD.

The last decade of research has pointed out the critical role of alteration of the circadian timing system in the disrupted BP circadian rhythm in subjects with metabolic and kidney diseases leading to subsequent development of CVD. Therefore, normalization of the disrupted BP rhythm by re-establishing circadian oscillation of the molecular components of the circadian timing system in both central and peripheral clocks is an important means of preventing the development of CVD. Recently, therapeutic strategies based on chronobiology, involving either the timing of drug administration or the use of novel pharmacological compounds that modify the circadian clock and the BP circadian rhythm, are becoming popular in clinical practice due to the observed meaningful outcomes.

### 6.1. Established Therapeutics Modulating the Dipping Pattern of BP Based on the Time of Administration

Accumulating evidence from clinical studies indicates that antihypertensive drugs are better taken in the evening, as nighttime BP strongly predicts CV events [129]. In a prospective, randomized, open-label, blinded end point trial in 448 hypertensive patients with T2D, Hermida et al. (2011) demonstrated that antihypertensive medications taken at bedtime produced a significantly lower CV risk and decreased the adjusted risk of CV death, myocardial infarction, and stroke [130]. The same group also demonstrated that the use of antihypertensive drugs at bedtime in patients with CKD caused a significant reduction in the risk of a composite outcome of CV disorders and death. Bedtime treatment significantly reduced mean sleep-time BP, conferring a 14% reduction in CV risk per 5-mmHg decrease in mean sleep-time BP [131]. Furthermore, a meta-analysis of 175 trials demonstrated that evening dosing of antihypertensive drugs as opposed to the usual dosing offered the greatest reduction in risk of CV events [132]. In an outstanding randomized cross-over trial, Bonten et al. (2014 and 2015) showed that intake of low-dose aspirin at bedtime compared with intake on awakening reduces morning platelet reactivity via a cyclooxygenase-1-dependent pathway [133], although they found in a follow-up study that aspirin intake at bedtime did not reduce 24-h ambulatory BP [134]. Regardless, this information is clinically significant, as CV events are most likely to occur in the early morning as compared to the day or night [135], and platelet reactivity likely contributes to this early morning peak [136]. Interestingly, a meta-analysis of 28 studies involving a total of 22,508 ESRD patients demonstrated a significant reduction in CV-related variables and left ventricular hypertrophy following nocturnal hemodialysis, compared with conventionally timed hemodialysis [137]. Systolic and diastolic BP, as well as mean arterial pressure results reveal significantly better responses to nocturnal hemodialysis, reflecting the results of a randomized control trial in which frequent nocturnal hemodialysis improved systemic BP and reduced left ventricular mass compared with conventionally timed hemodialysis [138], suggesting that nighttime dialysis is superior to daytime dialysis for lowering the risk of CVD. Obstructive sleep apnea has been shown to be an independent risk factor for hypertension in subjects with metabolic syndrome and associated CV disorders [139]. In patients with moderate-to-severe obstructive sleep apnea syndrome, nocturnal continuous positive airway pressure (CPAP) attenuates some of the adverse effects on the CV system [140], possibly through BP reduction [141].

### 6.2. Therapeutics Modulating the Circadian Timing System in Metabolic Disorders and Kidney Disease

#### 6.2.1. Melatonin

Re-establishing the circadian rhythm through pharmacological modification of the molecular components of the circadian timing system may ameliorate CV risk by improving the circadian rhythm of BP. Melatonin exhibits diurnal variation and plays an important role in the synchronization of the molecular circadian clocks in the SCN and peripheral tissues. Administration of melatonin reportedly reduces BP in both normotensive and hypertensive patients, and importantly, bedtime intake of melatonin reduces nighttime BP in essential hypertension through a direct hypothalamic effect, i.e., reduction of catecholamine levels, relaxation of vascular walls, and antioxidant effects [142,143,144]. Accumulating data have also confirmed the beneficial pharmacological effects of melatonin on abnormal function and tissue damage of the heart [145,146].

#### 6.2.2. Novel Compounds Targeting Molecular Components of the Circadian Timing System

Indeed, high-throughput screening has identified potent modulators of the molecular components of the circadian timing system, which inhibit the function of CKI inhibitors (CKIα/δ and CKIε/δ), thereby preventing the degradation of PER2 and significantly impacting the circadian oscillation of the core clock gene *PER* [147,148]. By contrast, Hirota et al. (2012) have demonstrated that KL001 acts as a stabilizer of CRY and inhibits glucagon-induced gluconeogenesis in primary hepatocytes [149]. The findings of this outstanding investigation suggest a possible future means of normalizing glucose homeostasis using KL001 in subjects with metabolic abnormalities. Recently, attention has turned to the generation of pharmacological compounds targeting the REV-ERB and ROR nuclear receptors to modulate the circadian timing system and associated physiological processes [150]. Solt et al. (2012) demonstrated that administration of REV-ERB agonists (SR9009 and SR9011, dual synthetic agonists of REV-ERBα and REV-ERBβ) alters the circadian behavior of mice and the circadian patterns of core clock gene expression in the hypothalamus and peripheral tissues [151]. Repeated treatment with these REV-ERB agonists in mice with diet-induced obesity led to a remarkable improvement in the metabolic profile by lowering plasma glucose, triglycerides, total cholesterol, non-esterified fatty acids, and leptin, as well as by dramatically reducing body weight and body fat mass. In line with these findings, Trump et al. (2013) optimized a series of REV-ERBα agonists based on GSK4112 and found that three of these compounds (No. 10, 16, and 23) exhibited similar pharmacokinetic and pharmacodynamic characteristics as SR9009, while another compound (No. 4) had an increased half-life and a bioavailability approximately 10 times greater than those of the other compounds [152]. As no in vivo data are yet available, the modulation of physiological processes by these compounds needs to be elucidated in animal models of metabolic dysfunction and CKD. By contrast, Kojetin et al. (2011) demonstrated that the REV-ERBα antagonist SR8278 stimulates the expression of the genes associated with gluconeogenesis [153]. However, a later study showed that SR8278 inhibits the activation of REV-ERBα by heme and GSK4112 along with glucose-induced insulin secretion in MIN-6 insulinoma cells, suggesting that this antagonist might be beneficial in subjects with hyperinsulinemia [154]. However, further in vivo studies are required to clarify the precise role of this antagonist in the modulation of the circadian clock, associated insulin homeostasis, and the non-dipping pattern of BP. Similarly, the RORα and RORγ agonist SR1078 has been reported to increase the expression of the RORα target gene *FGF*21 [155]. Of note, recombinant *FGF*21 improved glucose and triglyceride levels and insulin sensitivity in an animal model of diabetes [156]. Therefore, it can be speculated that SR1078 might improve glucose homeostasis, insulin sensitivity, and BP circadian rhythm in subjects with metabolic abnormalities. The potent RORα-specific inverse agonist SR3335, which was developed through the modification of previous agonists (SR1078 and T0901317), has satisfactory pharmacokinetic properties [157]. Moreover, in vivo administration of SR3335 dramatically improved glucose homeostasis by reducing Pck, a rate-limiting enzyme involved in gluconeogenesis. Taken together, all these findings suggest that pharmacological compounds targeting the REV-ERB and ROR nuclear receptors have the potential to improve glucose homeostasis and insulin sensitivity. However, extensive cutting-edge research should be undertaken to explore the precise molecular mechanisms underlying the beneficial effects of these pharmacological compounds and associated modification of the circadian rhythm of BP and CV disorders in subjects with metabolic dysfunction and CKD.

## 7. Conclusions

CVD remains a leading cause of death worldwide, and new approaches to the management and treatment of heart disease are clearly warranted and could benefit patients clinically. Extensive research in the field of chronobiology has revealed the critical roles of the molecular components of the circadian timing system in the development of CV disorders in connection with metabolic dysfunction and CKD. Furthermore, advanced research in this field has shown the effectiveness of chronotherapy, either by adjusting the timing of drug administration or by using novel pharmacological compounds that modulate the molecular components of the circadian timing system. Hence, the accumulating information summarized in this review may be helpful in the search for new molecular targets in terms of developing novel therapeutic strategies to prevent the onset and progression of CV disorders in subjects with metabolic disorders and CKD.

## Figures and Tables

**Table 1:** Altered expression profiles of molecular components of the circadian timing system in subjects with metabolic dysfunction that are directly or indirectly associated with a disrupted dipping pattern of BP.

Experimental Model	Molecular Mechanism	Altered Physiological Functions	References
*db*/*db* mice (type 2 diabetic model)	Attenuated circadian oscillation of *Bmal*1 and *Dbp*	Hypertension, disrupted BP circadian rhythm	Su et al. 2008 [17]
*db*/*db* mice	Altered clock gene expression both in central (*Per*1) and peripheral clock (*Per*1/2, *Cry*1/2), as well as their target genes (*Dbp*, *Ppar*γ, *Rev-erb*α)	Disrupted diurnal contraction of vasculature	Su et al. 2012 [18]
High-fat-diet-induced obesity in mice	Altered circadian oscillation of clock (*Per*1-3; *Cry*1/2; *Bmal*1) and clock-controlled (*Dbp*, *E*4*bp*4, *Pdk*4, *Pepck*, *Nhe*3) genes	Hyperglycemia, hypercholesterolemia, hyperinsulinemia	Hsieh et al. 2010 [19]
*db*/*db* mice	Altered rhythmic expression of *Per*2 and *Bmal*1, increased *Pai*-1 mRNA expression	Attenuated circadian behavior	Kudo et al. 2004 [20]
Streptozotocin (STZ)-induced diabetes in *Clock*-mutant mice	Circadian augmentation of *Pai*-1 is diminished	Clock is involved in the diabetes-induced circadian augmentation of *Pai*-1	Oishi et al. 2005 [21]
Dim light at night (alteration of circadian timing system) in mice	Attenuated circadian oscillation of *Per*1 and *Per*2 in hypothalamus, and *Rev-Erb* in liver and adipose tissue	Onset and progression of metabolic abnormalities	Fonken et al. 2011 [22]
Human subjects with type 2 diabetes (T2D)	*BMAL*1 single nucleotide polymorphisms (SNPs) (2 *BMAL*1 haplotypes)	SNP is associated with hypertension and T2D	Woon et al. 2007 [23]
Human subjects with T2D	*CLOCK*-rs4580704 (C > G) SNP	Cardiovascular (CV) disorders	Corella et al. 2016 [24]
Human subjects with rotating shiftwork	Interaction (epistatic effect) of serotonin transporter and *CLOCK* gene variation	Metabolic abnormalities	Sookoian et al. 2010 [25]
STZ-induced diabetes in mice	Altered circadian oscillation of core clock and their target genes	Contractile dysfunction of the heart	Young et al. 2002 [26]

**Table 2:** Altered expression profiles of the molecular components of the circadian timing system in subjects with CKD that are directly or indirectly associated with a disrupted dipping pattern of BP. (⬇) denotes reduced expression of indicated gene; (⬆) denotes increased expression of indicated gene.

Experimental Model	Molecular Mechanism	Altered Physiological Functions	References
5/6 nephrectomy in rats	Altered localization and diurnal variation of BMAL1, DBP, and PER2 in the kidney	Altered diurnal rhythm of renal function	Huang et al. 2013 [72]
Unilateral ureteral obstruction (UUO) in mice	*Agtrap* ⬇, *Usf* 1 ⬆, *Usf*2 ⬇	Renal dysfunction	Matsuda et al. 2013 [73]
5/6 nephrectomy in rats	Altered diurnal expression of clock genes (*Per*2, *Dbp*, and *Bmal*1), plasma renin activity, angiotensin II, and aldosterone	Severe kidney injury and altered diurnal rhythm of BP	Huang et al. 2013 [74]
5/6 nephrectomy in mice	*Dbp* ⬇, *Cyp*3a11 ⬇ and *Cyp*26a1 ⬇, *Tgf*-β1 ⬆	Altered hepatic metabolism aggravates renal dysfunction	Hamamura et al. 2016 [75]
UUO in Clock-KO mice	*Cyclooxygenase 2* ⬆, collagen synthesis ⬆, oxidative stress ⬆, *Tgf*-β ⬆	Renal dysfunction	Chen et al. 2015 [76]
5/6 nephrectomy in rats	Altered circadian oscillation of *Per*1 and 2 in the hypothalamus	Sleep disturbance associated with CKD	Hsu et al. 2012 [77]
